# The RNA-Binding Protein QKI Suppresses Cancer-Associated Aberrant Splicing

**DOI:** 10.1371/journal.pgen.1004289

**Published:** 2014-04-10

**Authors:** Feng-Yang Zong, Xing Fu, Wen-Juan Wei, Ya-Ge Luo, Monika Heiner, Li-Juan Cao, Zhaoyuan Fang, Rong Fang, Daru Lu, Hongbin Ji, Jingyi Hui

**Affiliations:** 1 State Key Laboratory of Molecular Biology, Shanghai Institutes for Biological Sciences, Chinese Academy of Sciences, Shanghai, China; 2 Shanghai Center for Plant Stress Biology, Shanghai Institutes for Biological Sciences, Chinese Academy of Sciences, Shanghai, China; 3 State Key Laboratory of Cell Biology, Institute of Biochemistry and Cell Biology, Shanghai Institutes for Biological Sciences, Chinese Academy of Sciences, Shanghai, China; 4 State Key Laboratory of Genetic Engineering, School of Life Sciences and Institutes for Biomedical Sciences, Fudan University, Shanghai, China; University of Michigan, United States of America

## Abstract

Lung cancer is the leading cause of cancer-related death worldwide. Aberrant splicing has been implicated in lung tumorigenesis. However, the functional links between splicing regulation and lung cancer are not well understood. Here we identify the RNA-binding protein QKI as a key regulator of alternative splicing in lung cancer. We show that QKI is frequently down-regulated in lung cancer, and its down-regulation is significantly associated with a poorer prognosis. QKI-5 inhibits the proliferation and transformation of lung cancer cells both *in vitro* and *in vivo*. Our results demonstrate that QKI-5 regulates the alternative splicing of *NUMB* via binding to two RNA elements in its pre-mRNA, which in turn suppresses cell proliferation and prevents the activation of the Notch signaling pathway. We further show that QKI-5 inhibits splicing by selectively competing with a core splicing factor SF1 for binding to the branchpoint sequence. Taken together, our data reveal QKI as a critical regulator of splicing in lung cancer and suggest a novel tumor suppression mechanism involving QKI-mediated regulation of the Notch signaling pathway.

## Author Summary

Alternative pre-mRNA splicing is a key mechanism for increasing proteomic diversity and modulating gene expression. Emerging evidence indicates that splicing program is frequently deregulated during tumorigenesis, and cancer cells favor to produce protein isoforms that can promote growth and survival. Lung cancer is one of the most common cancers and the leading cause of cancer-related death worldwide. Although a number of lung cancer-related splicing events have been detected in several genome-wide analyses, much less is known about how aberrant splicing takes place in lung cancer and how it contributes to tumor development. In this study, we characterized the RNA-binding protein QKI as a new critical regulator of alternative splicing in lung cancer and as a potential marker for prognosis. Genome-wide analysis of QKI-dependent splicing by RNA-Seq identified some cancer-associated splicing changes as its targets. Our results demonstrate that QKI-5 inhibits cancer cell proliferation and prevents inappropriate activation of the Notch signaling pathway by regulating its key target, *NUMB*. We further showed that QKI-5 represses the inclusion of *NUMB* alternative exon through competing with a core splicing factor SF1. In summary, our data indicate that down-regulation of QKI causes aberrant splicing in lung cancer and suggest a novel tumor suppression mechanism involving QKI-mediated repression of Notch signaling.

## Introduction

Lung cancer is one of the most common cancers and the leading cause of cancer-related death worldwide [1]. Due to the lack of detectable early-stage symptoms and limited treatment options, the 5-year survival rates remain poor for most patients [2]. Thus, more comprehensive investigations of gene expression alterations are needed for understanding the molecular mechanisms of lung tumorigenesis with a goal of identifying reliable earlier markers and effective therapeutic targets.

Alternative pre-mRNA splicing, the process by which multiple mRNA variants can be produced from a single gene, is a key mechanism for increasing proteomic diversity and modulating gene expression [3]. Misregulation of splicing underlies many human diseases, including cancer [4]–[6]. During the initiation and progression of cancer, the splicing program together with other layers of gene expression programs is subject to substantial alterations. A large fraction of RNA-binding proteins can function as splicing regulators to affect splice site selection through recognizing regulatory elements, located in either exons or introns, and interacting with spliceosomal factors or other splicing regulators [7]–[9]. The role of splicing regulators in cancer was best demonstrated by an SR protein, SRSF1, which functions as a proto-oncogene [10], [11]. Overexpression of SRSF1 resulted in the transformation of immortal fibroblasts and mammary epithelial cells partly through regulating alternative splicing of candidate genes involved in signal transduction and apoptosis. Other splicing regulators such as RBFOX2, hnRNP H, PTB have been shown to control cancer-associated splicing alterations that affect gene products participating in key cellular programs [12]–[14]. Many splicing factors exhibit differential expression between normal and tumor tissues [15], but their functions and targets during cancer development remain elusive.

Aberrant splicing has been implicated in lung tumorigenesis [16]. Indeed, a number of lung cancer-related splicing events have been detected in several genome-wide analyses using splicing sensitive microarray or deep sequencing technologies [17]–[21]. However, our understanding of the functionally important splicing events that contribute to tumorigenesis and the mechanisms that lead to aberrant splicing in lung cancer is very limited.

To search for splicing regulators that control lung-cancer associated splicing changes, we surveyed the expression of 59 known splicing regulators in microarray data collected from Gene Expression Omnibus (GEO) database [22]–[24] and found that the RNA-binding protein Quaking (QKI) is the most frequently down-regulated splicing factor in lung cancer tissues. QKI is a conserved STAR (signal transduction and activation of RNA) family protein that plays an essential role during embryonic and postnatal development [25]. QKI contains a STAR domain, which is composed of a maxi-KH RNA binding domain and two flanking QUA domains (QUA1 and QUA2), several SH3-binding sites, and a tyrosine-rich tail. The *QKI* gene encodes at least three protein isoforms (QKI-5, -6, and -7) that are generated by alternative splicing in mouse and human. QKI-5 isoform is a nucleus-cytoplasm shuttling protein, but found mostly in the nucleus [26]. QKI-6 and -7 isoforms are localized to the cytoplasm exclusively [27]. The expression of QKI proteins is developmentally regulated with high expression in adult lung, brain, heart, and testes [28], [29]. QKI proteins are involved in diverse aspects of RNA metabolism including pre-mRNA splicing [30]–[33], mRNA localization and transport [34], [35], mRNA and miRNA stability [35]–[40], translation [41], [42], and miRNA processing [43]. Based on SELEX and PAR-CLIP analyses, QKI has been shown to specifically bind an ACUAAY (Y = C or U) motif [44]–[46]. Recently, Hall et al showed that QKI-5 enhances *Capzb* exon 9 inclusion through recognizing ACUAA motifs downstream of exon 9 [32]. QKI-6 can also regulate alternative splicing in oligodendrocyte indirectly via controlling the translation or mRNA stability of a well-known splicing regulator, hnRNP A1 [39], [42]. However, the molecular mechanism underlying the direct role of QKI-5 in splicing regulation remains elusive.

In this study, we investigated the role of QKI during lung tumorigenesis. We found that QKI is frequently reduced in non-small cell lung cancer (NSCLC) and that its down-regulation is associated with shortened survival of patients. Through RNA-Seq analysis, we identified QKI as a master regulator of alternative splicing in lung cancer cells. Our results demonstrate that QKI inhibits lung cancer cell growth at least in part through regulating the alternative splicing of the Notch pathway regulator *NUMB*. Moreover, we show that QKI can inhibit splicing by selectively competing with a core splicing factor SF1 for targeting an authentic splicing signal, the branchpoint. Together, our data establish QKI as a critical regulator of splicing in lung cancer and present a new QKI/NUMB/Notch pathway in the regulation of cell proliferation.

## Results

### QKI is a prognostic marker that is frequently down-regulated in NSCLC

**Figure 1:**
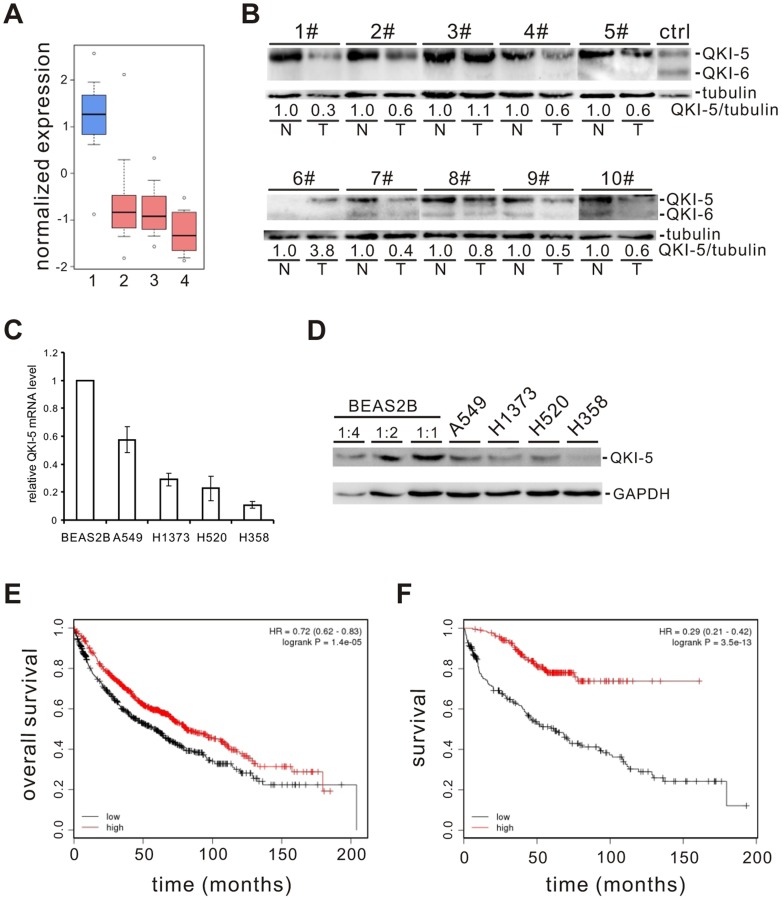
QKI is frequently down-regulated in lung cancer and associated with poorer cancer prognosis. (A) Down-regulation of *QKI* mRNA expression in patient samples of adenocarcinoma (2), squamous cell carcinoma (3), and large cell carcinoma (4) compared with that in the normal lung samples (1). Primary data were taken from the public database of Oncomine (http://www.oncomine.org). (B) Western blot analysis of QKI protein levels normalized to γ-tubulin using an anti-QKI antibody that recognizes all QKI isoforms in 10 paired adenocarcinoma tissues (T) and their adjacent normal tissues (N). HEK 293 cells overexpressing FLAG-tagged QKI-5 and QKI-6 were detected by anti-FLAG antibody indicating the positions of QKI-5 and QKI-6. (C, D) qRT-PCR (C) and Western blot (D) analyses of *QKI-5* mRNA and protein levels in a bronchial epithelial cell line BEAS2B and lung cancer cell lines A549, H1373, H520, and H358. (E, F) Kaplan-Meier survival curves for 1404 patients with NSCLC (E) or for 440 patients with stage I NSCLC (F). Primary data were taken from the Kaplan Meier plotter (http://www.kmplot.com).

To identify splicing factors that control lung cancer-associated splicing changes, we analyzed mRNA expression levels of 59 known splicing factors in microarray data collected from 80 normal and 104 adenocarcinoma patient samples [22]–[24] and found that QKI is one of the splicing factors frequently down-regulated (Figure S1). By surveying public gene expression databases (http://www.oncomine.org), we also observed that the expression levels of *QKI* mRNA are dramatically down-regulated in three major NSCLC subtypes, including adenocarcinomas, squamous cell carcinomas, and large cell carcinomas (Figure 1A). To validate it, we first examined the protein levels of QKI in primary lung cancer samples. In 8 out of 10 paired adenocarcinoma patient tissues, the expression level of QKI-5, the dominant isoform in lung cells, is significantly reduced in tumor tissues, compared to adjacent normal tissues (Figure 1B). We next found that both *QKI-5* mRNA and protein levels are down-regulated in a panel of human lung cancer cell lines, including A549, H1373, H520, and H358, compared with a bronchial epithelial cell, BEAS2B (Figure 1C and D). Importantly, using an online survival analysis tool (http://www.kmplot.com) [47], we found that *QKI* expression positively correlates with the overall survival of patients (Figure 1E). In particular, down-regulation of *QKI* in patients at stage I is significantly associated with a shortened survival rate (Figure 1F). These findings suggest that QKI may serve as an early-stage prognostic marker in the management of NSCLC.

### QKI-5 inhibits lung cancer cell proliferation and transformation

**Figure 2:**
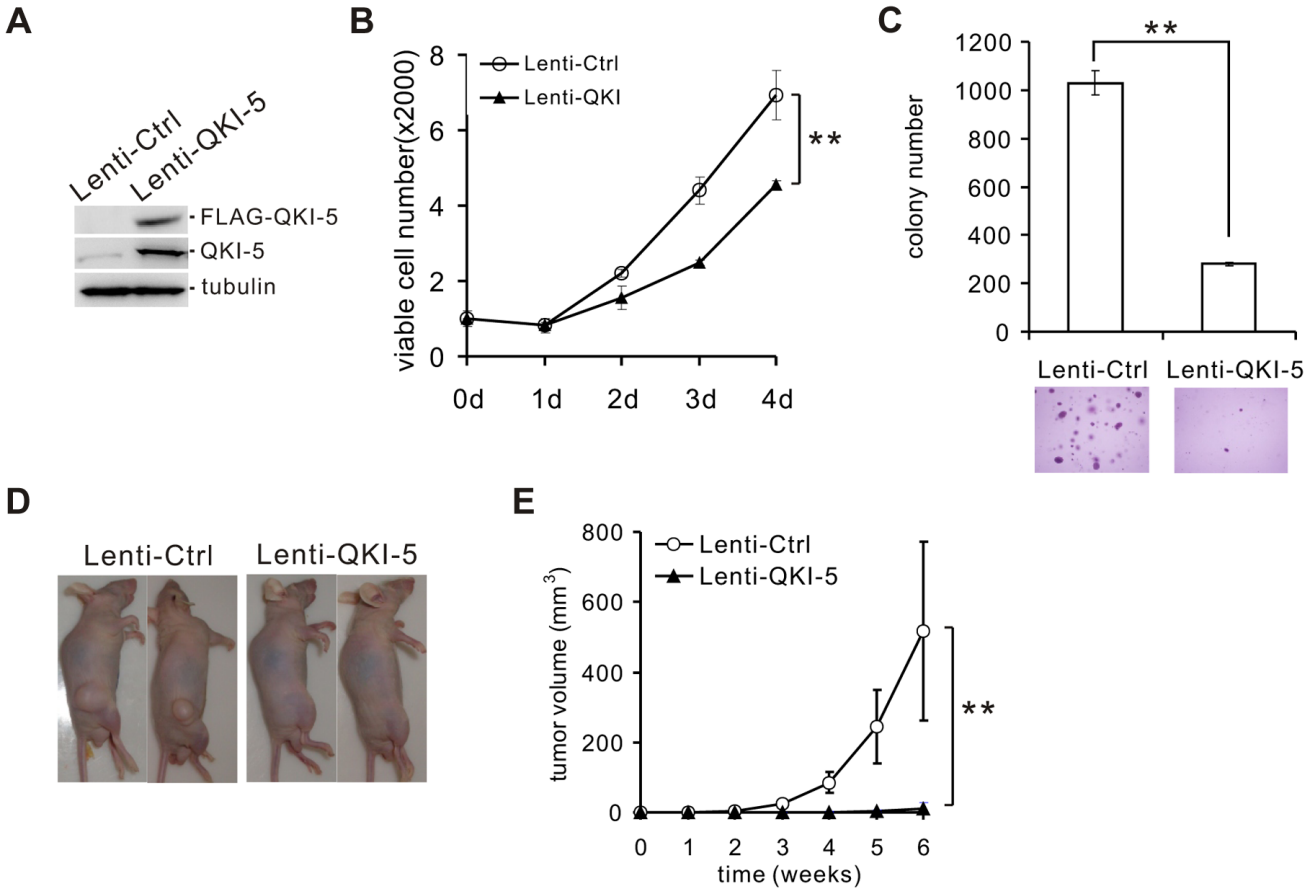
QKI-5 inhibits lung cancer cell proliferation and transformation. (A) Western blot analysis of QKI-5 expression in A549 cells stably transduced with a lentivirus vector (Lenti-Ctrl) or a FLAG-tagged QKI-5 expression construct (Lenti-QKI-5). (B) MTT analysis of cell proliferation in A549 cells described in A (** p<0.01, Student's t-test). Error bars represent standard deviations (n = 3). (C) Upper panel: quantification of colony formation on soft agar of A549 cells described in A (** p<0.01, Student's t-test). Error bars represent standard deviations (n = 3). Lower panels: representation of colonies visualized by microscopy. (D) A549 cells described in A were injected subcutaneously in nude mice. Representative images of treated mice are shown. (E) Tumor volume was measured weekly (** p<0.01, Student's t-test). Error bars represent standard deviations (n = 10).

To assess the role of QKI-5 in the pathogenesis of lung cancer, we made stable A549 and H520 cell lines overexpressing FLAG-tagged QKI-5 (Figure 2A and Figure S2A). Ectopic expression of QKI-5 in A549 and H520 cells showed a significantly slower proliferation than control cells as determined by the MTT assay (Figure 2B and Figure S2B). Furthermore, by performing anchorage-independent colony formation assay, we observed a dramatic decrease in the colony numbers from A549 and H520 cells overexpressing QKI-5 (Figure 2C and Figure S2C). These data indicate that QKI-5 is capable of inhibiting both cell proliferation and transformation *in vitro*.

To determine whether QKI-5 suppresses tumor growth *in vivo*, we injected A549 cells stably expressing FLAG-tagged QKI-5 into nude mice (n = 10, each group). Six weeks after injection, 100% of mice injected with the control A549 cells formed tumors. In contrast, only 30% of mice injected with A549 cells expressing exogenous QKI-5 generated tumors. Additionally, these tumors were much smaller in volume (Figure 2D and E). We conclude that QKI-5 inhibits cancer cell growth *in vitro* and *in vivo*.

### QKI is a key regulator of alternative splicing in lung cancer cells

**Figure 3:**
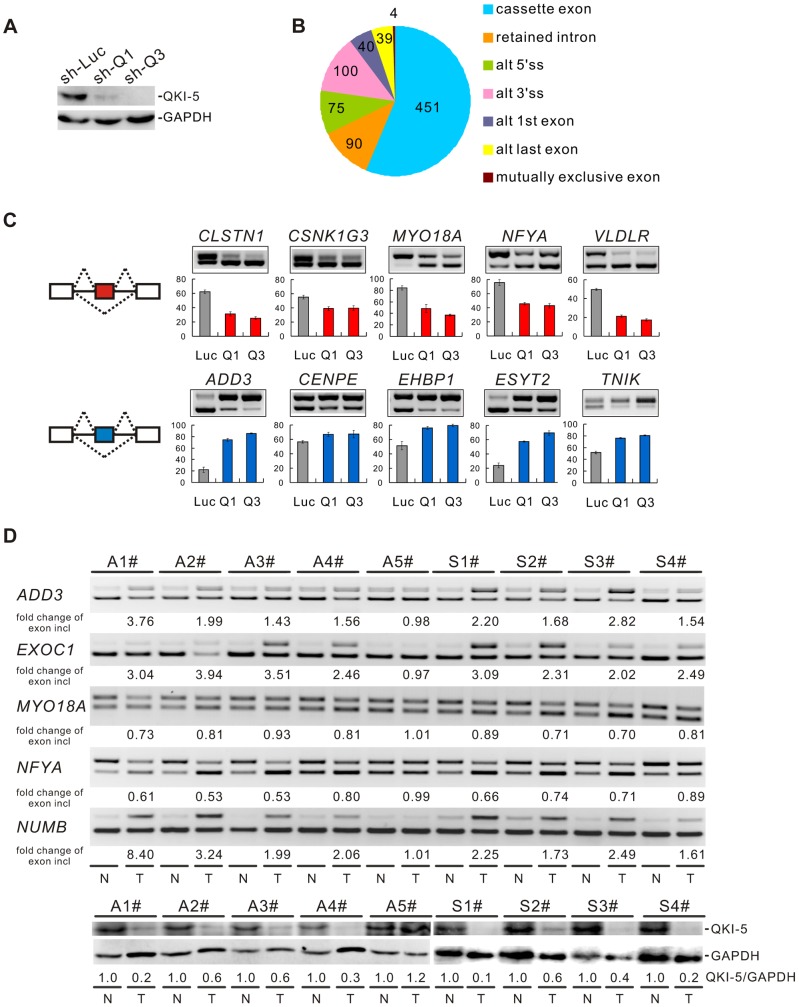
QKI regulates cancer-associated splicing changes. (A) Western blot analysis of QKI-5 expression in BEAS2B cells stably expressing control (sh-Luc) and two QKI shRNAs (sh-Q1 and sh-Q3). (B) RNA-Seq analysis detected 799 events with alternative splicing changes after QKI was knocked down (Benjamini-Hochberg adjusted *P*-value<0.05). (C) RT-PCR analysis of the splicing patterns of QKI targets in control- and QKI-knockdown BEAS2B cells. The average percentages of exon inclusion with standard deviations are shown below (n = 3). (D) Down-regulation of QKI in NSCLC causes cancer-associated splicing events. Fold changes of exon inclusion in tumor tissue normalized to that in normal tissue are shown below the representative RT-PCR analyses. A1–A5: adenocarcinoma patient samples. S1–S4: squamous cell carcinoma patient samples. Among the 9 paired samples, the expression levels of QKI-5 protein normalized to GAPDH are down-regulated in all paired samples except A5 patient samples as determined by Western blotting. T: tumor tissue; N: adjacent normal tissue.

To understand the molecular basis for QKI function in lung cancer, we performed a genome-wide search for mRNA targets of QKI. To do this, QKI was depleted from BEAS2B cells using retroviruses expressing shRNAs targeting all three QKI isoforms (Figure 3A). Total RNAs were then isolated from control- (sh-Luc) or QKI-knockdown (sh-Q3) cells followed by sequencing using an Illumina GAII sequencer. In total, 78.4 and 76.5 million reads were generated for control- and QKI-knockdown samples, respectively; and ∼73% of reads were mapped to the human genome (hg19 version) uniquely. Using this method, we identified 213 genes whose mRNAs exhibited an over 2-fold change in abundance after QKI knockdown (RPKM> = 0.5). We also detected 799 events of splicing changes in 629 genes (Benjamini-Hochberg adjusted *P*-value<0.05, Figure 3B and Table S1). Among them, only 3 alternatively spliced genes had an over 2-fold change in the mRNA level, suggesting that the mRNA abundance and alternative splicing of most QKI targets are not co-regulated. To validate the RNA-Seq data, we performed semi-quantitative RT-PCR analyses for a subset of cassette exon-type splicing events. Among the 90 tested cassette exon events, 81 events (90%) displayed changes in splicing upon QKI depletion in BEAS2B cells, confirming our RNA-Seq results (Table S2). As representatives, we show 10 validated targets for which QKI functions either as a splicing activator or as a splicing repressor (Figure 3C).

As described above, we have found that QKI is down-regulated in NSCLC, which correlates with a poorer prognosis. To investigate the significance of QKI-mediated splicing regulation in cancer, we examined approximately 40 splicing events from Table S2 in tumor tissues in comparison with matching normal tissues from the same patients. RT-PCR analyses detected at least 25 alternative splicing events that underwent changes in the same direction as shown in BEAS2B cells upon QKI knockdown (Table S3). For example, the splicing patterns of *ADD3*, *EXOC1*, *MYO18A*, *NFYA*, and *NUMB* were changed in 4 pairs of adenocarcinoma (A1-4#) and 4 pairs of squamous cell carcinoma tissues (S1-4#), in which the expression levels of QKI are significantly reduced in tumor tissues compared to the adjacent normal tissues. However, the splicing patterns of these genes were not changed in 1 pair of adenocarcinoma tissues (A5#) in which comparable amount of QKI was detected between tumor and normal tissues (Figure 3D). These results suggest that down-regulation of QKI in NSCLC causes cancer-related splicing events.

### QKI suppresses the Notch pathway through regulating the alternative splicing of *NUMB*

**Figure 4:**
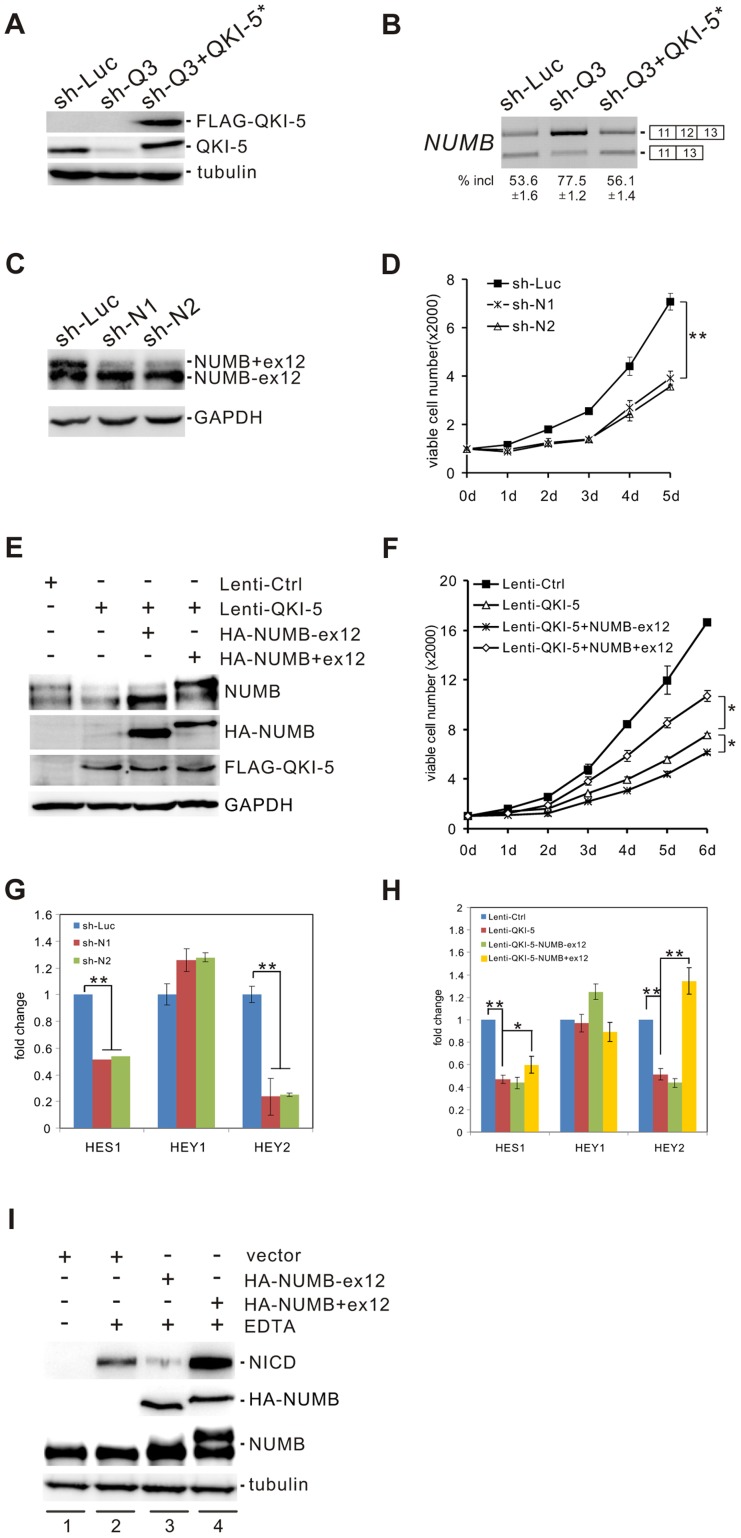
QKI-5 regulates *NUMB* splicing and the Notch signaling pathway. (A) Western blot analysis of QKI-5 expression in BEAS2B cells stably transduced with retroviruses expressing control shRNA (sh-Luc), QKI shRNA (sh-Q3) or QKI shRNA together with a QKI-resistant construct (sh-Q3+QKI-5*). (B) RT-PCR analysis of the splicing patterns of *NUMB* in BEAS2B cells described in A. The average percentages of exon inclusion with standard deviations are shown below (n = 3). (C) Western blot analysis of NUMB expression in A549 cells stably transduced with retroviruses expressing control shRNA (sh-Luc) and two shRNAs (sh-N1 and sh-N2) specifically target the exon 12 of *NUMB*. (D) MTT analysis of cell proliferation in A549 cells described in C (** p<0.01, Student's t-test). Error bars represent standard deviations (n = 3). (E) The two *NUMB* isoforms were transfected into A549 cells stably expressing QKI-5. The expression of NUMB and QKI-5 was determined by Western blotting. (F) MTT analysis of cell proliferation in A549 cells described in E (* p<0.05, Student's t-test). Error bars represent standard deviations (n = 3). (G) Real-time qRT-PCR analysis of the expression of Notch targets, *HES1*, *HEY1*, and *HEY2* in A549 cells described in C (** p<0.01, Student's t-test). Error bars represent standard deviations (n = 3). (H) Real-time qRT-PCR analysis of the expression of Notch targets, *HES1*, *HEY1*, and *HEY2* in A549 cells described in E (* p<0.05, ** p<0.01, Student's t-test). Error bars represent standard deviations (n = 3). (I) C2C12 cells were transiently transfected with a vector (lanes 1, 2) or HA-tagged NUMB expression constructs (lanes 3, 4) in the absence (lane 1) or the presence (lanes 2–4) of EDTA treatment. The expression levels of NICD and NUMB were detected by Western blotting.

NUMB is an evolutionarily conserved signaling adaptor protein that plays a critical role in cell fate determination and functions as a negative regulator of the Notch signaling pathway [48]–[50]. The human *NUMB* gene generates four isoforms (p65, p66, p71, and p72) that result from the alternative splicing of two variable exons [51]. Exon 6 encodes 11 amino acids within the phosphotyrosine binding (PTB) domain, while exon 12 encodes 48 amino acids within the C-terminal proline-rich region (PRR). Interestingly, we found that *NUMB* is a target of QKI. Knockdown of QKI in BEAS2B cells stimulated exon 12 inclusion, and re-introduction of QKI-5 in QKI-depleted cells repressed exon 12 inclusion (Figure 4A and B). In contrast, QKI-5 did not regulate the splicing of exon 6 in *NUMB* (Figure S3).

To investigate the significance of QKI regulation on *NUMB* alternative splicing, we designed two shRNAs (sh-N1 and sh-N2) targeting different regions of *NUMB* exon 12. Knockdown of the exon 12-containing isoforms of NUMB in A549 cells significantly decreased cell proliferation (Figure 4C and D). Furthermore, introduction of a NUMB isoform containing exon 6 and exon 12 (p72) into A549 cells ectopically expressing QKI-5 partially abrogated the anti-proliferation activity of QKI-5. In contrast, expression of a NUMB isoform with exon 6 but without exon 12 (p66) augmented the inhibitory effect of QKI-5 on cell proliferation (Figure 4E and F). Thus, our data strongly suggest that QKI-5 suppresses cell proliferation, at least in part, through controlling the alternative splicing of *NUMB*.

To determine whether alternative splicing of *NUMB* regulates the Notch pathway, we examined the expression of the Notch targets, *HES1*, *Hey1*, and *Hey2*, by RT-qPCR analysis after isoform-specific knockdown of NUMB in A549 cells. Removal of the NUMB isoforms containing exon 12 resulted in a 2- and 5-fold decrease of *HES1* and *Hey2* mRNA levels, respectively. However, the same treatment had little effect on *Hey1* expression (Figure 4G). We next tested whether QKI also regulates the expression of these Notch targets through changing the splicing pattern of *NUMB*. Overexpression of QKI-5 in A549 cells inhibited *HES1* and *Hey2* expression, but did not affect *Hey1* expression. Importantly, re-expression of the *NUMB* isoform p72 in QKI-5-overexpressing cells fully rescued *Hey2* expression, while it slightly increased *HES1* expression (Figure 4H). In contrast, introduction of the *NUMB* isoform p66 in QKI-5-overexpressing cells did not increase, but slightly decreased the expression of *HES1* and *Hey2*. Together, these results indicate that QKI represses the expression of Notch targets through inhibiting the inclusion of *NUMB* exon 12.

To understand how the alternative splicing of *NUMB* regulates the Notch pathway, we investigated the ability of the two NUMB isoforms p72 and p66 to activate the Notch receptor in C2C12 cells, which express endogenous Notch1 receptor at a relatively high level. To do this, the released intracellular fragment of Notch (NICD) was detected after transiently transfecting C2C12 cells with the NUMB isoforms followed by EDTA treatment. As previously reported, production of the active form of Notch receptor (NICD) was stimulated in C2C12 upon EDTA treatment [52] (Figure 4I, lanes 1 and 2). Remarkably, NICD was not detected when the NUMB isoform p66 was overexpressed in C2C12 cells. In contrast, overexpression of the NUMB isoform p72 enhanced NICD release (Figure 4I, lanes 3 and 4). These data indicate that the two isoforms of NUMB have opposite effects on the Notch signaling activation, i.e. the NUMB isoform without exon 12 inhibits NICD activation, whereas the NUMB isoform carrying exon 12 promotes NICD activation. Taken together, our data demonstrate that QKI-5 suppresses the activation of the Notch pathway through regulating the alternative splicing of *NUMB*.

### QKI-5 inhibits the inclusion of *NUMB* exon 12 by recognizing two QKI binding sites located upstream of and within exon 12

**Figure 5:**
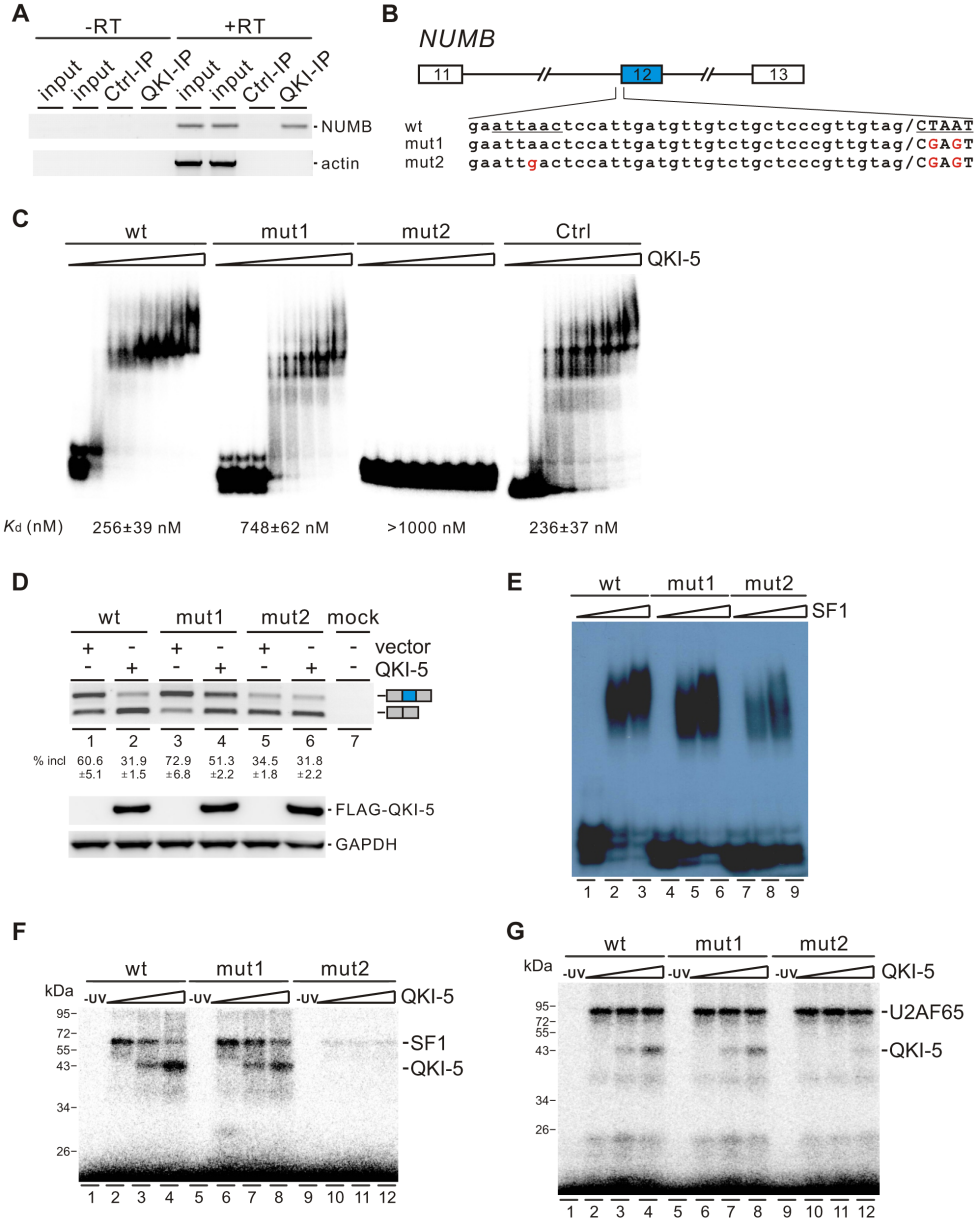
QKI-5 inhibits the inclusion of *NUMB* exon 12 by recognizing two binding sites flanking the 3′ splice site of intron 12. (A) *NUMB* pre-mRNA is associated with QKI *in vivo*. DNA fragments amplified from *NUMB* pre-mRNA and *β-actin* mRNA immunoprecipitated with a control mouse IgG or a QKI antibody were detected by RT-PCR analysis. Minus RT reactions were performed to exclude the possibility of DNA contamination. (B) Schematic representation of NUMB wildtype and mutant constructs. Intron sequences are in small, exon sequences in capital, and slashes indicate exon/intron junctions. (C) Gel shift analysis of QKI-5 binding to *NUMB* pre-mRNAs. ^32^P-labeled short RNAs containing the last 56 nt of intron 12 and the first 25 nt of exon 12 were incubated with increasing concentrations (0, 130, 260, 390, 520, 780, 1040, and 2080 nM) of recombinant His-tagged QKI-5. A control RNA carrying a bipartite QKI consensus sequence defined previously by SELEX [45] served as a positive control. RNA-protein complexes were fractionated on a 5% native polyacrylamide gel. (D) RT-PCR analysis of the splicing patterns of wildtype and mutant minigene constructs transfected into HEK 293 cells together with a vector or a QKI-5 expression construct. The average percentages of exon inclusion with standard deviations are shown below (n = 3). As a control, no DNA was transfected (mock). The positions of splicing products are shown on the right. (E) Gel shift analysis of SF1 binding to *NUMB* pre-mRNAs. ^32^P-labeled RNAs as described in B were incubated with 0, 5, and 10-fold molar excess of recombinant GST-tagged SF1 (aa 1–361). RNA-protein complexes were fractionated on a 5% native polyacrylamide gel. (F, G) UV crosslink analyses of QKI-5, SF1 or U2AF65 binding to *NUMB* pre-mRNAs. ^32^P-labeled RNAs as described in B were UV-crosslinked in the presence of 5-fold molar excess of recombinant GST-tagged SF1 (aa 1–361) (F) or 10-fold molar excess of recombinant GST-tagged U2AF65 (G) supplemented with 0, 5 and 10-fold molar excess of purified His-tagged QKI-5, followed by SDS-PAGE. As a control, a reaction without UV irradiation was performed (-UV).

To understand how QKI regulates *NUMB* exon 12 splicing, we first checked whether QKI is associated with *NUMB* pre-mRNA *in vivo* by performing RNA immunoprecipitation (RIP) assay. Our data showed that QKI antibody specifically immunoprecipitated *NUMB* pre-mRNA but not the *β-actin* mRNA (Figure 5A). Next, we analyzed carefully the pre-mRNA sequence of *NUMB*. In the regions surrounding the 3′ splice site of intron 12, we found two QKI binding sites (AUUAAC and CUAAU), which are similar to the previously defined QKI binding consensus ACUAAY (Y = C or U) [44]–[46]. We made minigene constructs containing exons 11, 12, and 13 together with two shortened introns flanking exon 12. The second site or both sites were mutated to generate mut1 and mut2 constructs (Figure 5B). We then assayed the QKI binding affinities of the wildtype and mutant RNAs containing the last 56 nt of intron 12 and the first 25 nt of exon 12 sequences by a gel shift assay. The binding affinity of wildtype RNA to QKI is comparable to that of a control RNA, which carries a bipartite QKI consensus sequence determined previously using the SELEX method [45]. Compared with wildtype RNA, mut1 RNA exhibited reduced QKI-5 binding, while mut2 RNA lost QKI-5 binding completely (Figure 5C). This result indicates that both sites bind QKI-5. To determine whether QKI-5 inhibits exon 12 splicing through recognizing these two elements, we transfected *NUMB* minigene constructs together with a vector or a QKI-5 expression construct into HEK 293 cells. Overexpression of QKI-5 significantly inhibited exon 12 inclusion of the wildtype minigene (Figure 5D, lanes1 and 2). Mutation of the second site within exon 12 (mut1) increased exon 12 inclusion, indicating that the second site acts a silencing element for exon 12 inclusion. Ectopically expressed QKI-5 was still able to repress exon 12 inclusion, although the repression effect was less than that on the wildtype minigene (Figure 5D, lanes 3 and 4). This is not surprising, because QKI still binds but less efficiently to mut1 RNA compared to the wildtype RNA (Figure 5C). Mutation of the two sites resulted in a decrease of exon 12 inclusion (Figure 5D, lane 5). This is probably due to reduced binding of SF1 to mut2 pre-mRNA (Figure 5E, lanes 7–9). Interestingly, overexpression of QKI-5 had little effect on exon 12 inclusion of mut2 minigene (Figure 5D, lane 6). These data indicate that QKI inhibits *NUMB* exon 12 inclusion by recognizing two QKI binding sequences flanking the 3′ splice site of intron 12.

Since the first binding site (AUUAAC) is localized upstream of the polypyrimidine tract of intron 12 and since it is similar to the SF1 consensus ACUNAC (N = any nucleotides) sequence [53], we tested the SF1 binding activity of the wildtype and mutant RNAs by a gel shift assay. SF1 bound efficiently to the wildtype and mut1 RNAs, but the A to G mutation in mut2 RNA led to the reduction of SF1 binding (Figure 5E). We have shown that both SF1 and QKI bind to the first site (AUUAAC) upstream of the 3′ splice site (Figure 5C and E). Thus, our results suggest that QKI may repress exon 12 inclusion by competing with SF1 binding. We tested this idea by performing UV crosslink competition assay. QKI-5 inhibited SF1 binding to the wildtype RNA, but to a lesser extent for the mut1 RNA (Figure 5F, lanes 2–4 and 6–8). QKI-5 did not compete the weak SF1 binding to the mut2 RNA (Figure 5F, lanes 10–12). These data indicate that the two QKI binding sites may collaborate to enhance binding of one another, which explains why QKI competes less effectively with SF1 on the mut1 RNA. In addition, we show that QKI-5 did not affect U2AF65 binding to the wildtype and mutant RNAs (Figure 5G). Together, our results indicate that QKI-5 recognizes two binding sites located upstream of and within exon 12, and represses the inclusion of *NUMB* exon 12 by competing with SF1.

### QKI-5 can inhibit splicing by selectively competing with SF1 for binding to the branchpoint

**Figure 6:**
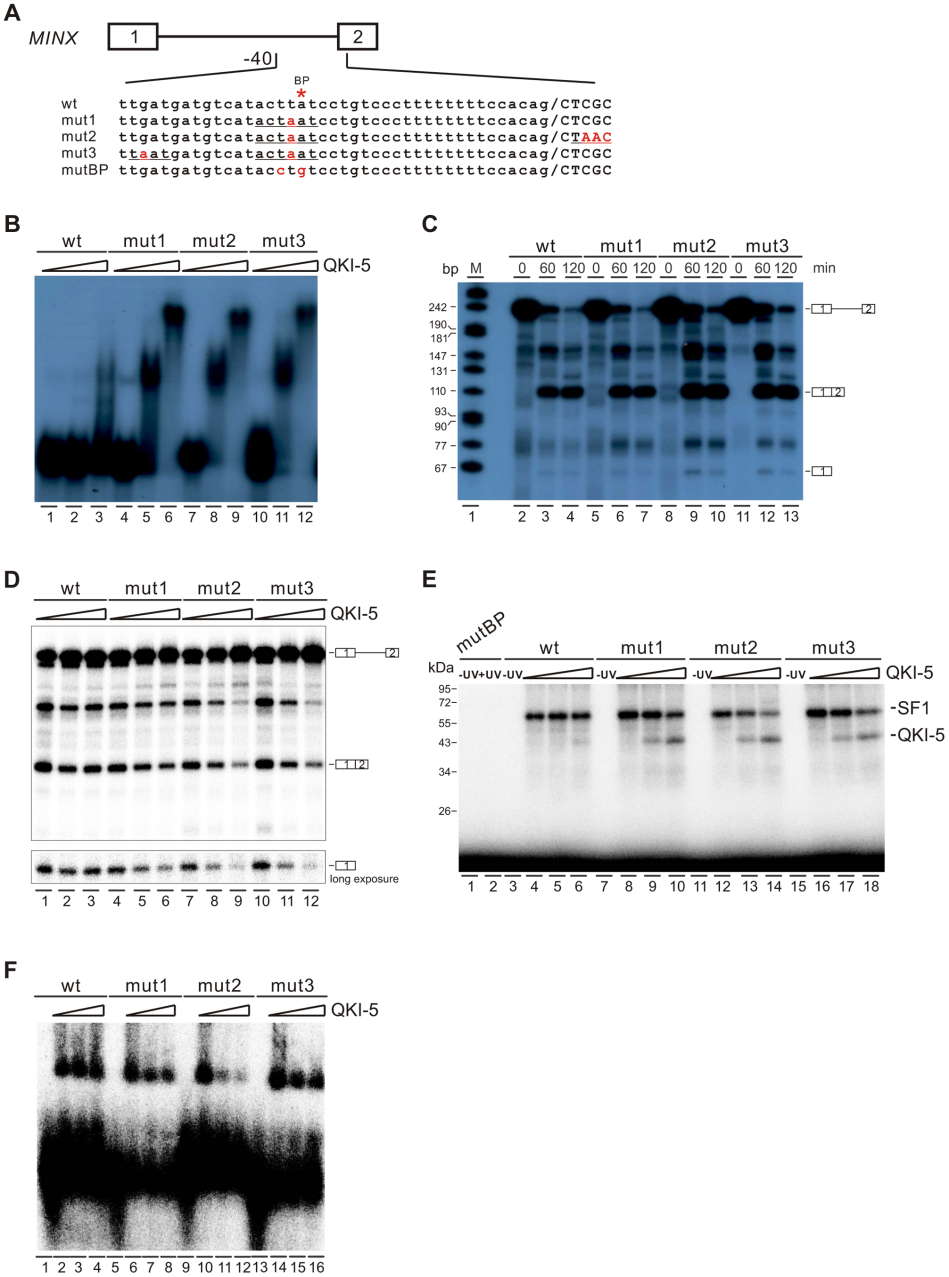
QKI-5 represses splicing by selectively competing with SF1. (A) Schematic representation of MINX wildtype and mutant constructs. (B) Gel shift analysis of QKI-5 binding to MINX pre-mRNAs. ^32^P-labeled short RNAs containing the 3′ half of the intron and the second exon were incubated with 0, 20, and 40-fold molar excess of recombinant His-tagged QKI-5. RNA-protein complexes were fractionated on a 5% native polyacrylamide gel. (C) *In vitro* splicing of wildtype and mutant MINX pre-mRNAs. ^32^P-labeled pre-mRNAs were spliced in HeLa cell nuclear extract for the time indicated. The positions of pre-mRNA, first exon and spliced products are indicated on the right. M, ^32^P-labeled pcDNA3 HpaII restriction fragments. (D) *In vitro* splicing of wildtype and mutant MINX pre-mRNAs in the HeLa cell nuclear extract supplemented with different amounts of recombinant His-tagged QKI-5 (0, 0.5 and 1 µM). (E) UV crosslink analysis of QKI-5 or SF1 binding to MINX full-length pre-mRNAs. ^32^P-labeled wildtype and mutant MINX pre-mRNAs were UV-crosslinked in the presence of 15-fold molar excess of recombinant GST-tagged SF1 (aa 1–361) supplemented with 0, 15 and 30-fold molar excess of purified His-tagged QKI-5, followed by SDS-PAGE. As a control, a reaction without UV irradiation was performed (-UV). (F) A complex assembly on the wildtype and mutant pre-mRNAs. ^32^P-labeled wildtype and mutant MINX pre-mRNAs containing the 3′ half of the intron and the second exon were incubated with HeLa nuclear extract supplemented with different amounts of purified His-tagged QKI-5 (0, 0.2 and 0.4 µM). Complexes were fractionated on a 1.5% low melting agarose gel.

To gain further mechanistic insights into QKI-mediated splicing repression, we tested directly whether QKI inhibits splicing by competing with SF1 for targeting the branchpoint in an *in vitro* splicing system. We mutated the branchpoint sequence (ACUUAU, where the branchpoint adenosine is underlined) in MINX reporter pre-mRNA to the QKI binding consensus ACUAAU [54] (Figure 6A, mut1). Since it has been shown previously that QKI binds to the ACUAAY consensus, which is often accompanied by a so-called half site UAAY in close proximity [45], we also introduced a UAAU or UAAC site upstream or downstream of the ACUAAU motif (Figure 6A, mut2 and mut3). In the gel shift analysis, the wildtype MINX RNA bound poorly to the purified QKI-5. Mut2 and mut3 RNAs bound QKI more efficiently than mut1 at a low concentration of QKI-5, and all three RNAs formed larger RNA-protein complexes at a high concentration of QKI-5 (Figure 6B). These results indicate that the A4 in the consensus motif ACUAAY is crucial for QKI binding.

We next tested whether these mutations affect MINX splicing in HeLa nuclear extract with or without supplemented QKI-5 (Figure 6C and D). In HeLa nuclear extract alone, the splicing activity of mut1, mut2 and mut3 is comparable to that of wildtype (Figure 6C). This is likely due to the low abundance of QKI in HeLa nuclear extract. Addition of purified QKI-5 to the HeLa nuclear extract did not affect the splicing of wildtype MINX pre-mRNA, but inhibited the splicing of mut1, mut2 and mut3 pre-mRNAs (Figure 6D). Moreover, the result shows that QKI-5 inhibits splicing before the first step of splicing reaction, since production of the first exon was reduced after QKI-5 addition to the HeLa nuclear extract.

To determine whether QKI-5 affects the splicing of mutant pre-mRNAs by competing SF1 binding to branchpoint sequence, we conducted UV crosslink experiment. SF1 did not recognize a pre-mRNA in which the branchpoint sequence is destroyed (mutBP, Figure 6A), while SF1 interacted with the wildtype, mut1, mut2, and mut3 pre-mRNAs (Figure 6E). Notably, QKI-5 significantly inhibited SF1 binding to mut1, mut2, and mut3 pre-mRNAs, but had nearly no effect on SF1 binding to wildtype pre-mRNA (Figure 6E). Since the recognition of branchpoints by SF1 is essential for A complex formation, we constructed pre-mRNA substrates containing the 3′ half of MINX intron and the second exon and analyzed A complex assembly on these substrates. Addition of QKI-5 to the HeLa nuclear extract inhibited A complex assembly on the mutant pre-mRNAs, but had no effect on the wildtype pre-mRNA (Figure 6F). Thus, we conclude that QKI-5 can inhibit splicing by selectively competing with SF1 for binding to the branchpoint sequence.

## Discussion

Emerging evidence indicates that splicing program is frequently deregulated during tumorigenesis, and cancer cells favor to produce protein isoforms that can promote growth and survival. However, how aberrant splicing takes place and how it contributes to tumor development are not well understood. In this study, we identified the RNA-binding protein QKI-5 as a new critical regulator of alternative splicing in NSCLC and as a potential marker for prognosis. Furthermore, we show that QKI-5 regulates the alternative splicing of *NUMB*, thereby inhibiting cancer cell proliferation and preventing the activation of the Notch signaling pathway. Our findings suggest a novel tumor suppression pathway and expand our understanding of splicing regulation in tumorigenesis.

**Figure 7:**
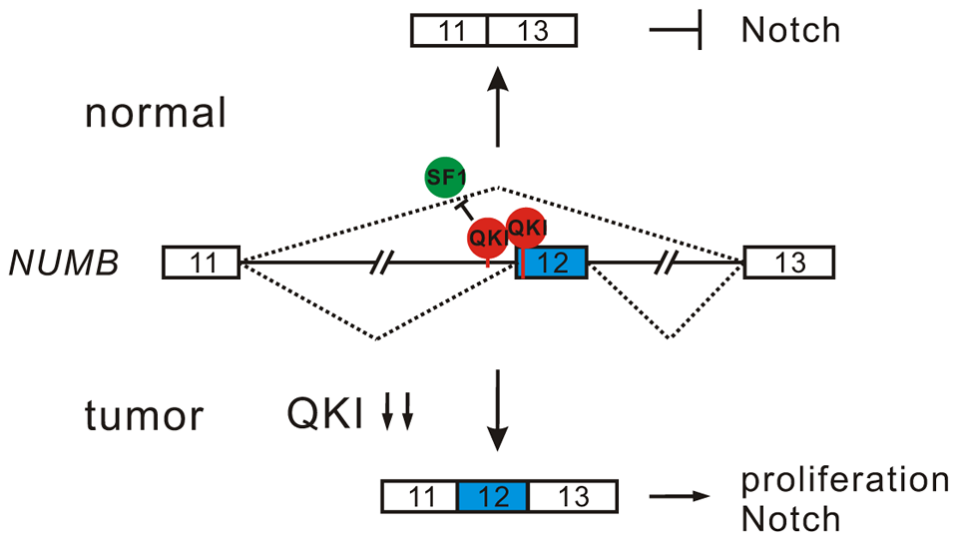
A model for QKI-mediated control of cell proliferation. In normal cells, QKI-5 recognizes two elements in *NUMB* pre-mRNA and inhibits exon 12 inclusion by competing with SF1. In tumor cells, down-regulation of QKI leads to the expression of NUMB isoform containing exon 12, which is able to activate Notch signaling and cell proliferation.

Inappropriate activation of Notch signaling has been linked to a wide range of cancers including glioblastoma, colon, pancreatic, breast, and lung tumors [55], [56]. However, the molecular mechanisms of aberrant Notch activation in cancers are not well understood. In breast and lung cancers, there is frequently loss of NUMB-mediated suppression of Notch signaling due to ubiquitination-induced degradation of all NUMB protein isoforms [57], [58]. The authors of these two studies also noticed that when substantial levels of NUMB were detected in some NSCLC patient specimens, the Notch pathway was still highly activated. This implicates that additional mechanisms may lead to the dysregulation of NUMB-mediated repression of Notch signaling. Increased inclusion of *NUMB* exon 12 has been reported in several types of cancer including lung, ovarian, and breast cancers [12], [19], [20]. A previous study suggests that differential expression of NUMB isoforms has different effects on the Notch signaling [20]. However, how *NUMB* alternative splicing is regulated in cancer had remained unclear. Here, we show that alternative splicing of *NUMB* is regulated by QKI-5, and that this regulation is critically important for the control of cell proliferation (Figure 4E and F). Furthermore, our data clearly indicate that the two NUMB isoforms p72 and p66 have the opposite effects on the activation of the Notch pathway (Figure 4I). It is possible that NUMB isoform carrying exon 12, which encodes additional 48 aa in the protein-protein interaction domain PRR, may recruit other factors to stimulate NICD activation, or it may behave as a dominant negative form to antagonize the function of the NUMB isoform with shorter PRR domain. In summary, our results suggest a new QKI/NUMB/Notch pathway in the control of cell proliferation (Figure 7). Taken together that the alternative splicing of *NUMB* exon 12 is developmentally regulated [59]–[62] and that QKI is essential for embryonic development, it suggests that the QKI/NUMB/Notch pathway identified in this study may play a role during development.

In this work, we also show that QKI can either positively or negatively regulate exon inclusion (Figure 3C). To gain mechanistic insights into QKI-mediated splicing regulation, we generated an RNA map of QKI based on its known binding consensus sequence ACUAAY and our RNA-Seq data. This RNA map shows that QKI often activates exon inclusion when its binding sites are located in the downstream intron regions of the regulated exons, and represses exon inclusion when its binding sites are located within regulated exons or in the upstream intron regions (Figure S4). This indicates that QKI regulates alternative splicing in a position-dependent manner. The position-dependent effect of splicing regulators has emerged as a key mechanism in splicing regulation. QKI shares the same positional principles with several other splicing regulators including Nova, Fox1/2, and MBNL [63]–[66]. Recently, Hall et al showed that QKI depletion in myoblasts induces widespread alternative splicing changes and exhibits similar aforementioned positional effects on splicing regulation [32]. However, so far the mechanisms underlying the position-dependent splicing regulation remain to be investigated. A recent large-scale analysis of splicing lariats reveals that most human branchpoints are located between 18 and 35 nt upstream of the 3′ splice site [67]. In the QKI RNA map we generated, we noted that a peak of ACUAAY motif, which represents the potential branchpoint, is located within 40 nt upstream of the two 3′ splice sites in the randomly selected control pre-mRNAs (Figure S4). Remarkably, ACUAAY motif is significantly enriched within 40 nt upstream of QKI-repressed exons. This observation suggests that QKI binding sites overlap with the branchpoints upstream of QKI-repressed exons. During the initial step of spliceosome assembly, the branchpoint is first recognized by SF1. Both SF1 and QKI contain the KH and QUA2 domains which contribute to the sequence-specific RNA recognition. The optimal binding site of human SF1 (ACUNAC, where the branchpoint adenosine is underlined) is very similar to the QKI binding consensus (ACUAAY). Using minigene and *in vivo* splicing assay, we showed that QKI-5 inhibits the inclusion of *NUMB* exon 12 by competing with SF1 (Figure 5). In addition, although the RNA recognition motifs of SF1 and QKI are similar, we found that they display differential binding affinities to RNA elements containing similar sequences. Using *in vitro* reporter assay, we showed that QKI-5 can inhibit splicing by selectively competing with a core splicing factor SF1 for binding to the branchpoint (Figure 6). These findings suggest that QKI plays a vital role in fine-tuning the activity of SF1.

This study and those of others strongly suggest that QKI functions as a tumor suppressor [40], [68]. However, the functional targets of QKI in cancers are largely unknown. Our genome-wide analysis of QKI targets by RNA-Seq established QKI-5 as a master regulator of alternative splicing in lung cancer cells (Figure 3B). Down-regulation of QKI in NSCLC causes a number of cancer-associated splicing events (Figure 3D and Table S3). Beside *NUMB*, we also found other critical targets of QKI that have been previously implicated in tumorigenesis. For example, BIN1, a nucleocytoplasmic adaptor protein, functions as a tumor suppressor that directly binds and inhibits c-Myc. As shown in Figure S5, QKI-5 in BEAS2B cells induced the exclusion of *BIN1* exon12A, which has been shown to abolish its binding to c-Myc and lose its anti-oncogenic activity in melanoma cells [69]. Since down-regulation of QKI is significantly associated with poor prognosis at early cancer stages (Figure 1F), it indicates that QKI and its targets identified in this study may serve as earlier markers for lung cancer. We also show that increasing QKI-5 levels in lung cancer cells suppresses cell proliferation and transformation both *in vitro* and *in vivo*, implicating that QKI is a potential drug target for the cancer treatment. In summary, our study demonstrates that QKI is a critical regulator of aberrant splicing in lung cancer and that the Notch signaling regulator, *NUMB*, is a key target of QKI in the control of cell proliferation.

## Materials and Methods

### Oligonucleotides

The sequences of all the oligonucleotides used in this study are listed in Table S4.

### Plasmid construction

Plasmid construction is described in Protocol S1.

### Ethics statement

All paired lung normal and tumor tissues were collected from informed, consenting patients in Fudan University Shanghai Cancer Center (Shanghai, China) from October 2007 to February 2012 with approval from the Institute Research Ethics Committee. All mice were treated according to the protocols approved by the Institutional Animal Care and Use Committee of the Shanghai Institutes for Biological Sciences, Chinese Academy of Sciences.

### Cell culture

The human bronchial epithelial BEAS2B cells were grown in Dulbecco's Modified Eagle's medium supplemented with 10% fetal bovine serum. The human lung cancer A549, H1373, H520 and H358 cells were cultured in RPMI-1640 medium supplemented with 10% fetal bovine serum.

### Real-time quantitative reverse-transcription polymerase chain reaction (qRT-PCR)

Total RNA was extracted from patient tissues and cultured cells using Trizol (Invitrogen, USA) and reverse transcribed into first-strand cDNA from random hexamers using MMLV reverse transcriptase (Promega, USA). PCR was then performed using SYBR Green PCR Master Mix (Applied Biosystems, USA) on a 7500 Fast Real-Time PCR system according to manufacturer's instruction (Applied Biosystems, USA).

### Lentivirus production and infection

To generate recombinant lentivirus, HEK 293T cells were transfected with lentiviral expression constructs together with two helper plasmids pVSVG and delta-R-8.2 using a calcium phosphate method. A549 and H520 cells were infected by recombinant lentiviruses and screened for stable expression of QKI-5 and NUMB in the presence of puromycin according to manufacturer's instruction (System Biosciences, USA).

### Western blotting

To extract the protein from patient samples, normal and tumor tissues were homogenized in RIPA buffer containing 50 mM Tris-Cl pH 7.4, 150 mM NaCl, 1% NP-40, 1 mM EDTA-free protease inhibitor cocktail (Roche, Germany) plus 1.5 mM phenylmethylsulfonyl fluoride (PMSF). Lysates were collected following the removal of insoluble material from tissue extracts by centrifugation at 14,000 rpm for 20 min at 4°C. Protein lysates were separated by sodium dodecyl sulfate-polyacrylamide gel electrophoresis (SDS-PAGE) followed by gel transfer to a nitrocellulose membrane (BioRad, USA). The membranes were incubated first with the primary antibodies, and then with secondary antibodies coupled to horseradish peroxidase (HRP). Band signals were detected with an enhanced chemiluminescence (ECL) system (Thermo Scientific) and visualized by image analyzer (Fujifilm, Japan). The primary antibodies used for this study are anti-GAPDH (Kang Cheng Bio-tech, China), anti-QKI (Sigma, USA), anti-γ-tubulin (Sigma, USA), anti-FLAG (Sigma, USA), anti-NUMB (Cell Signaling, USA), anti-NICD (Val1744, Cell Signaling, USA), and anti-HA (Roche, Germany). The HRP-conjugated secondary antibodies anti-mouse IgG and anti-rabbit IgG were purchased from Promega.

### MTT cell proliferation assay

Cells were seeded at a density of 2000/well in 24-well culture plates. After 24, 48, 72, 96, and 120 h of incubation, cells were treated with 3-(4, 5-methylthiazol-2-yl)-2, 5-diphenyltetrazolium bromide (MTT) with a final concentration of 5 µg/ul for 4 h. The resulting formazan was solubilized with dimethylsulfoxide (DMSO), and the absorption was measured at 570 nm using a spectrophotometer (Thermo Scientific, USA). The cell viability was expressed as the optical density at 570 nm.

### Anchorage-independent soft agar colony assay

Cells were plated at a density of 10,000/well in a top layer of RPMI-1640 medium containing 10% FBS and 0.4% low melting point agar (Invitrogen, USA) which is over a bottom layer of RPMI-1640 medium containing 10% FBS and 1% low melting point agar. Each cell line was plated in triplicates. After 4 weeks, cells were stained with 0.5 ml crystal violet overnight. The colonies were counted from 3 random fields under an inverted microscope.

### Tumorigenicity assay

Six-week-old male nude mice were injected subcutaneously with 1×10^6^ cells of A549 stable cell lines. Tumor growth was monitored by caliper measurement once a week for at least 6 weeks. The tumor volume (cm^3^) was calculated using the following formula: 0.5× (L×W^2^), where L is tumor length and W is tumor width. Six weeks after inoculation, the mice were sacrificed by CO_2_ asphyxiation.

### Activation of Notch1

C2C12 cells were transfected with a vector and two NUMB cDNA constructs using Lipofectamine 2000 reagent (Invitrogen, USA). 48 h after transfection, C2C12 cells were washed with PBS twice, and incubated for 15 min with pre-warmed PBS containing 5 mM EDTA. Treated cells were then washed with PBS and chased for 30 min in regular culture medium.

### Protein purification

*E. coli* BL21 DE3 cells transformed with expression plasmids encoding GST-tagged SF1 (aa 1–361), GST-tagged U2AF65, and His-tagged QKI-5 were induced with 0.3 mM isopropyl beta-D-1-thiogalactopyranoside (IPTG) for 5 h at 37°C. Purification of GST- and His-tagged proteins was performed using glutathione-Sepharose 4B or Ni-NTA according to manufacturer's instructions (GE Healthcare, USA; QIAGEN, Germany).

### *In vitro* splicing

*In vitro* splicing was performed as previously described [70]. Briefly, a 25 µl of splicing reaction was setup by incubating 10 ng of ^32^P-labeled RNA substrates with HeLa nuclear extract (Cil Biotech, Belgium) at 30°C.

### RNA immunoprecipitation (RIP)

BEAS2B cells were harvested and lysed in lysis buffer (50 mM Tris-HCl, pH 7.4, 100 mM NaCl, 1% NP-40, 0.1% SDS, 0.5% sodium deoxycholate, 1/100 protease inhibitor cocktail from Roche). After centrifuging at 10,000 g for 10 min, an aliquot (10%) of supernatant was removed and served as input. The remaining supernatant was immunoprecipitated with either a mouse IgG or a QKI antibody immobilized on Protein G Sepharose. The bound RNAs were washed extensively and isolated using TRIzol (Invitrogen, USA) and reverse transcribed into first-strand cDNA using random hexamers and MMLV reverse transcriptase (Promega, USA). PCRs were then performed using primers T7-NUMB-for/rev for detecting *NUMB* pre-mRNA and ACTB-for/rev for detecting *β-actin* mRNA. The resulting DNA fragments were separated on a 2% agarose gel and visualized by ethidium bromide staining.

### Gel shift assay

DNA templates for *in vitro* transcription were obtained by PCR using primer sets T7-MINX-For/MINX-2, T7-NUMB-For/-Rev, and T7-NUMB-For/-mut-Rev. 10 ng of ^32^P-labeled RNAs were incubated with different molar excess of recombinant His-tagged QKI-5 and GST-tagged SF1 (aa 1–361) protein under standard splicing conditions at 30°C for 20 min. An aliquot of 10 µl was removed and incubated with 2 µl of tRNA (1 mg/ml) for 5 min. RNA-protein complexes were fractionated by a 5% native polyacrylamide gel and visualized with a phosphoimager (Fujifilm, Japan).

### UV crosslinking

20 ng of ^32^P-labeled RNAs were incubated with recombinant His-tagged QKI-5 in the absence or presence of GST-tagged SF1 (aa 1–361) and U2AF65 under standard splicing conditions at 30°C for 20 min. UV crosslinking was done on ice for 20 min with 254-nm UV light. Unprotected RNAs were removed by RNase A at 37°C for 20 min. Crosslinked species were analyzed on a 12.5% SDS polyacrylamide gel and visualized with a phosphoimager (Fujifilm, Japan).

### *In vivo* splicing

The day before transfection, 2×10^5^ BEAS2B cells were seeded in 35 mm culture dishes. Minigene plasmids were transfected using Lipofectamine 2000. 24 h after transfection, total RNAs were isolated using Trizol Reagent. The first-strand cDNAs were reverse- transcribed using BGH-rev as primer and MMLV reverse-transcriptase following the manufacturer's instruction. PCR was performed using target gene-specific primers.

### Spliceosome assembly

To prepare RNA substrates for A complex assembly, DNA templates for *in vitro* transcription were obtained by PCR using primer set T7-MINX-For/MINX-2. 10 ng of ^32^P-labeled RNA substrate was incubated in HeLa cell nuclear extract supplemented with different amount of recombinant His-tagged QKI-5 protein under standard splicing condition for 15 min. 5 µl of the reaction was removed and mixed with 1 µl of heparin (5 mg/ml) and 1 µl of glycerol (87%) to stop the reaction. The samples were separated by a 1.5% low melting agarose gel. After drying the gel, signals were visualized with a phosphoimager (Fujifilm, Japan).

### RNA-Seq and data analysis

Total RNAs isolated from BEAS2B cells expressing a control shRNA or a QKI shRNA were subjected to single-end RNA-Seq using Illumina GAII platform according to the manufacturer's instruction. We performed RNA-Seq twice with read lengths 50 nt and 75 nt, individually.

Data analysis was carried out according to a previous publication [71]. Briefly, we first obtained the annotations of human protein coding genes from Ensembl database and generated splicing-junctions by joining every exon with all possible downstream exons within the same gene. To force an alignment overhang of at least 6 nt from one side of the junction to the other, the exon sequences on either side of the junction were 44 and 69 nt for 50 and 75 nt reads, respectively. Secondly, the RNA-Seq reads were aligned to the human genome (hg19) and the splicing-junctions by Bowtie allowing for up to three mismatches. The ambiguously aligned reads were excluded from further analysis. The junctions that were covered by at least two independent reads were reported. Thirdly, we searched for the alignments that gave the evidence of seven types of alternative splicing events: cassette exon (SE), retained intron (IR), alternative 5′ splicing site (A5SS), alternative 3′ splicing site (A3SS), mutually exclusive exon (MXE), alternative first exon (AFE), and alternative last exon (ALE). To identify the significantly changed alternative splicing events, a Fisher's exact test was performed for each event using a 2*2 contingency table consisting of the read counts from either the inclusion or exclusion isoforms in the control and treated sample. The RNA-Seq data are available at NCBI's GEO database under the accession number GSE55215.

## Supporting Information

Figure S1 Heat map of mRNA expression levels for splicing regulators in adenocarcinoma patient samples. The mRNA expression levels of 59 known splicing regulators in 104 adenocarcinoma tissues are compared to 80 normal tissues. The microarray data were downloaded from Gene Expression Omnibus (GEO) database with accession number: GSE10799, GSE7670, and GSE10072.
(PDF)

Figure S2 QKI-5 inhibits cell proliferation and transformation in H520 cells. (A) Western blot analysis of QKI-5 expression in H520 cells stably transduced with a lentivirus vector (Lenti-Ctrl) or a FLAG-tagged QKI-5 expression construct (Lenti-QKI-5). (B) MTT analysis of cell proliferation in H520 cells described in A (** p<0.01, Student's t-test). Error bars represent standard deviations (n = 3). (C) Upper panels: quantifications of colony formation on soft agar of H520 cells described in A (p<0.01, Student's t-test). Error bars represent standard deviations (n = 3). Lower panels: representations of colonies visualized by microscopy.
(PDF)

Figure S3 QKI-5 does not affect alternative splicing of *NUMB* exon 6. RT-PCR analysis of the splicing pattern of *NUMB* in BEAS2B cells stably transduced with retroviruses expressing control shRNA (sh-Luc), QKI shRNA (sh-Q3) or QKI shRNA together with a QKI-resistant construct (sh-Q3+QKI-5*). The asterisk indicates a non-specific PCR product.
(PDF)

Figure S4 QKI regulates alternative splicing in a position-dependent manner. The numbers of ACUAA(U/C) motifs in the pre-mRNAs from 244 QKI-activated cassette exons (red curves) and 207 QKI-repressed cassette exons (blue curves) are mapped. The alternative exons are shown in gray box and constitutive exons in black. The green curves represent the average numbers of ACUAA(U/C) motifs in control pre-mRNAs which are not regulated by QKI. Error bars indicate the 99.9999% confidence.
(PDF)

Figure S5 QKI-5 regulates the alternative splicing of *BIN1*. RT-PCR analysis of the splicing patterns of *BIN1* in BEAS2B cells stably transduced with retroviruses expressing control shRNA (sh-Luc), QKI shRNA (sh-Q3) or QKI shRNA together with a QKI-resistant construct (sh-Q3+QKI-5*). The determination of endogenous and exogenous QKI-5 expression is shown in Figure 4A. The positions of splicing products are shown on the right.
(PDF)

Protocol S1 Supplementary methods for plasmid construction and the generation of QKI RNA map.
(DOC)

Table S1 Alternative splicing changes detected upon QKI knockdown in BEAS2B cells by RNA-Seq.
(XLS)

Table S2 Validated QKI targets.
(XLS)

Table S3 Down-regulation of QKI causes lung cancer-associated alternative splicing changes.
(XLS)

Table S4 Sequences of all oligonucleotides used.
(XLS)
