# Supplementary figures and images for: The RNA-Binding Protein QKI Suppresses Cancer-Associated Aberrant Splicing
Source: PLoS Genet. 2014 Apr 10;10(4):e1004289. doi: 10.1371/journal.pgen.1004289 (PMC3983035; doi:10.1371/journal.pgen.1004289)

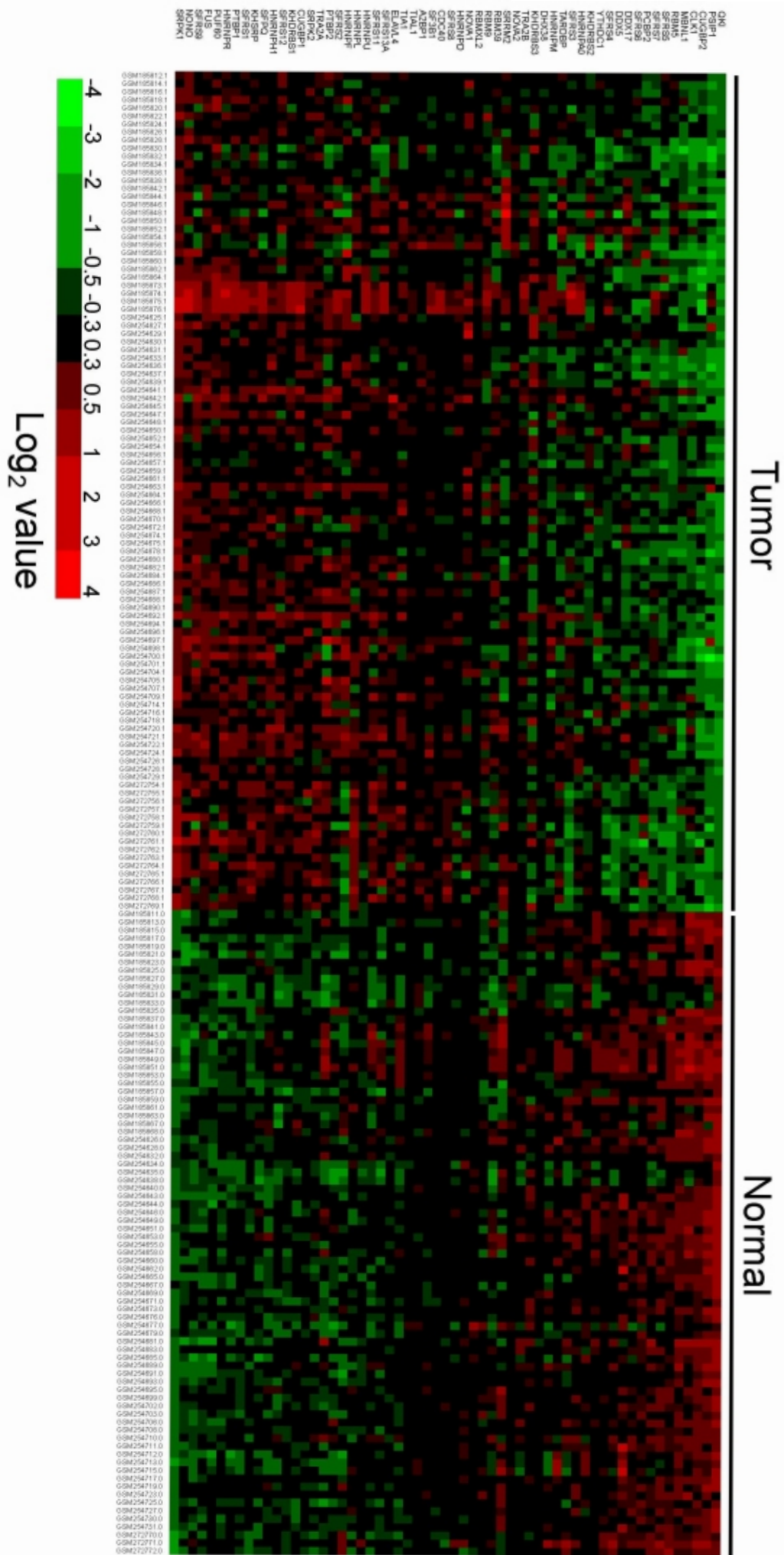

Supplement: Figure S1 — Heat map of mRNA expression levels for splicing regulators in adenocarcinoma patient samples. The mRNA expression levels of 59 known splicing regulators in 104 adenocarcinoma tissues are compared to 80 normal tissues. The microarray data were downloaded from Gene Expression Omnibus (GEO) database with accession number: GSE10799, GSE7670, and GSE10072. (PDF) [file pgen.1004289.s001.pdf]

**A**

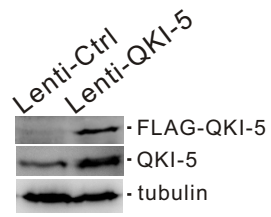

**B**

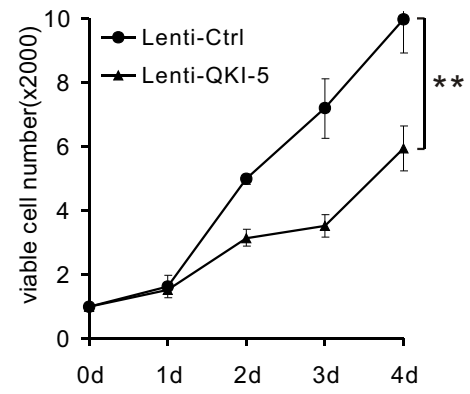

**C**

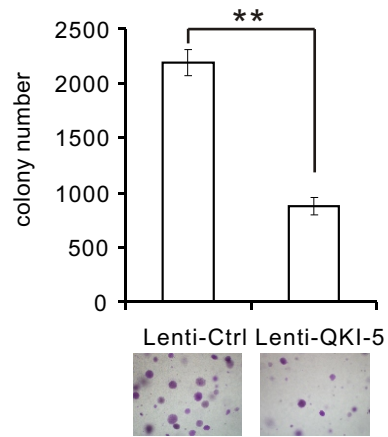

Supplement: Figure S2 — QKI-5 inhibits cell proliferation and transformation in H520 cells. (A) Western blot analysis of QKI-5 expression in H520 cells stably transduced with a lentivirus vector (Lenti-Ctrl) or a FLAG-tagged QKI-5 expression construct (Lenti-QKI-5). (B) MTT analysis of cell proliferation in H520 cells described in A (** p<0.01, Student's t-test). Error bars represent standard deviations (n = 3). (C) Upper panels: quantifications of colony formation on soft agar of H520 cells described in A (p<0.01, Student's t-test). Error bars represent standard deviations (n = 3). Lower panels: representations of colonies visualized by microscopy. (PDF) [file pgen.1004289.s002.pdf]

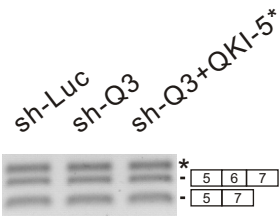

Supplement: Figure S3 — QKI-5 does not affect alternative splicing of NUMB exon 6. RT-PCR analysis of the splicing pattern of NUMB in BEAS2B cells stably transduced with retroviruses expressing control shRNA (sh-Luc), QKI shRNA (sh-Q3) or QKI shRNA together with a QKI-resistant construct (sh-Q3+QKI-5*). The asterisk indicates a non-specific PCR product. (PDF) [file pgen.1004289.s003.pdf]

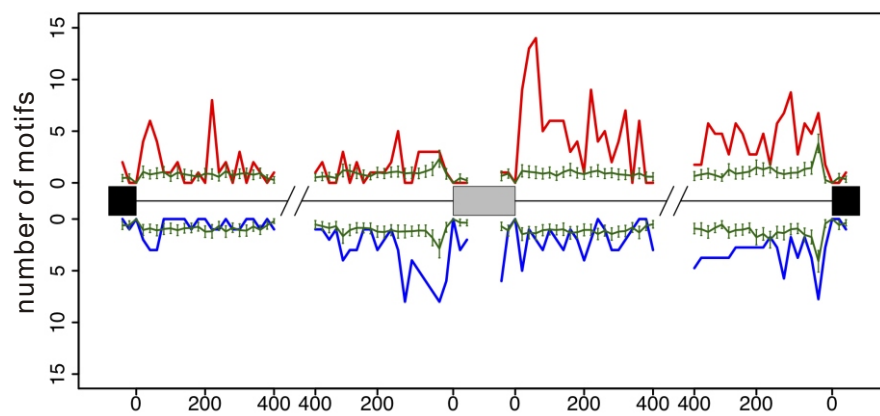

Supplement: Figure S4 — QKI regulates alternative splicing in a position-dependent manner. The numbers of ACUAA(U/C) motifs in the pre-mRNAs from 244 QKI-activated cassette exons (red curves) and 207 QKI-repressed cassette exons (blue curves) are mapped. The alternative exons are shown in gray box and constitutive exons in black. The green curves represent the average numbers of ACUAA(U/C) motifs in control pre-mRNAs which are not regulated by QKI. Error bars indicate the 99.9999% confidence. (PDF) [file pgen.1004289.s004.pdf]

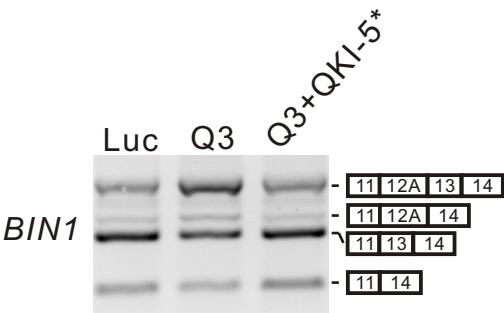

Supplement: Figure S5 — QKI-5 regulates the alternative splicing of BIN1. RT-PCR analysis of the splicing patterns of BIN1 in BEAS2B cells stably transduced with retroviruses expressing control shRNA (sh-Luc), QKI shRNA (sh-Q3) or QKI shRNA together with a QKI-resistant construct (sh-Q3+QKI-5*). The determination of endogenous and exogenous QKI-5 expression is shown in Figure 4A. The positions of splicing products are shown on the right. (PDF) [file pgen.1004289.s005.pdf]
