# Supplementary material for: The RNA-Binding Protein QKI Suppresses Cancer-Associated Aberrant Splicing
Source: PLoS Genet. 2014 Apr 10;10(4):e1004289. doi: 10.1371/journal.pgen.1004289 (PMC3983035; doi:10.1371/journal.pgen.1004289)
Supplement: Protocol S1 — Supplementary methods for plasmid construction and the generation of QKI RNA map. (DOC) [file pgen.1004289.s006.doc]

**Plasmid construction**

QKI-5 expression constructs

The cDNA fragment encoding the full-length QKI-5 with a 3’ FLAG epitope was amplified using primers QKI-5-for1 and QKI-5-FLAG-rev1 and inserted between BamHI and XbaI sites of the pcDNA3 vector (Invitrogen, USA). The same cDNA fragment was amplified using primers QKI-5-for2 and QKI-5-FLAG-rev2 and subcloned between XbaI and BamHI sites of the lentiviral vector pCDH-CMV-MCS-EF1-Puro (System Biosciences, USA). To construct the plasmid expressing His-tagged QKI-5, the cDNA fragment was amplified using primer pair QKI5-SalI-for/QKI5-HindIII-rev and inserted between the SalI and HindIII sites of the pET-28a-c(+) vector (Novagen, USA).

NUMB expression constructs

The cDNA fragments encoding the full-length NUMB isoforms p72 and p66 with a 3’ HA tag were amplified using primers NUMB-for1 and NUMB-HA-rev1 and inserted between XbaI and NotI sites of the lentiviral vector pCDH-CMV-MCS-EF1-Puro (System Biosciences, USA).

SF1 expression construct

The cDNA fragment encoding the N-terminal part of SF1 (aa 1-361) was amplified using primer pair SF1-BamHI-for/SF1-SalI-rev and inserted between the BamHI and SalI sites of the vector pGEX-5X-2 (GE Healthcare, USA).

shRNA expression constructs

Retroviral plasmids containing human QKI and NUMB exon 12 shRNA target and control sequences were made by annealing oligonucleotides sh-Q1-F/sh-Q1-R, sh-Q3-F/sh-Q3-R, sh-N1-F/sh-N1-R, sh-N2-F/sh-N2-R and sh-Luc-F/sh-Luc-R followed by ligation into the EcoRI and BamHI sites of pSIREN-RetroQ (Clontech, USA).

Minigene constructs for *in vivo* splicing

All minigene constructs consist of three exons and two introns. The genomic sequences of the minigene unit were amplified by using genomic DNA isolated from HEK 293 cells as template. To construct the wild type *NUMB* minigene, most of the intron sequences was deleted from 300 nt downstream of the 5’ ss to 300 nt upstream of the 3’ ss, PCRs were performed using oligonucleotides NUMB-1 to -6 as primers. A complete fragment was assembled by an overlap PCR, using the previous PCR products as templates, and inserted into between BamHI and XhoI sites of pcDNA3. In the mutant minigene constructs, QKI binding sites in the wild type minigene constructs were mutated using a similar overlap PCR mutagenesis method and oligonucleotides NUMB-mut-1 to -4.

MINX constructs

To generate MINX mut1, mut3 and mutBP constructs, pSP6-MINX was used as a template for a two-step PCR. Primer pairs MINX-1/-5 and MINX-2/-4 were used for cloning the mut1 construct, MINX-1/-11 and MINX-2/-10 for the mut3 construct, and MINX-1/-mutBP-Rev and MINX-2/-mutBP-For for the mutBP construct. To generate the MINX mut2 construct, mut1 plasmid was used as a template, and MINX-1/-7 and MINX-2/-6 as primers for a two-step PCR. The PCR fragments were then inserted between EcoRI and BamHI sites of pSP6-MINX to substitute the wildtype fragment.

**Generation of QKI RNA map**

The numbers of QKI motif were counted in 40 nt exonic region or 400 nt intronic region surrounding the 5’ or 3’ splice sites with a 20 nt bin for 244 QKI-activated and 207 QKI-repressed events, respectively. For the short exon (< 80 nt) and intron (< 800 nt), we divided the exon or intron sequence into half. To count the numbers of QKI motif for control pre-mRNAs, we randomly selected the same amount of detected cassette-exon events which are not regulated by QKI (p>0.95). We repeated this procedure 100 times and calculated the average numbers with 99.9999% confidence interval for each bin.
